# Effects of the COVID-19 pandemic on the rates of adverse birth outcomes and fetal mortality in Japan: an analysis of national data from 2010 to 2022

**DOI:** 10.1186/s12889-024-18905-z

**Published:** 2024-05-28

**Authors:** Tasuku Okui, Naoki Nakashima

**Affiliations:** Medical Information Center, Kyushu University Hospital, Maidashi3-1-1 Higashi-ku Fukuoka city Fukuoka prefecture, Fukuoka city, 812-8582 Japan

**Keywords:** Japan, Birth, COVID-19, Fetal mortality

## Abstract

**Background:**

Although the coronavirus disease 2019 (COVID-19) pandemic affected trends of multiple health outcomes in Japan, there is a paucity of studies investigating the effect of the pandemic on adverse birth outcomes and fetal mortality. This study aimed to investigate the effect of the onset of the pandemic on the trends in adverse birth outcomes and fetal mortality using national data in Japan.

**Methods:**

We used the 2010–2022 birth and fetal mortality data from the Vital Statistics in Japan. We defined the starting time of the effect of the pandemic as April 2020, and the period from January 2010 to March 2020 and that from April 2020 to December 2022 were defined as the pre- and post- pandemic period, respectively. The rates of preterm birth, term low birth weight (TLBW), small-for-gestational-age (SGA), large-for-gestational-age (LGA), spontaneous fetal mortality, and artificial fetal mortality were used as outcomes. An interrupted time series analysis was conducted using monthly time series data of the outcomes to evaluate the effects of the pandemic. In addition, a modified Poisson regression model was used to evaluate the effects of the pandemic on these outcomes using individual-level data, and the adjusted risk ratio of the effect was calculated.

**Results:**

The adverse birth and fetal mortality outcomes showed a decreasing trend over the years, except for preterm birth and LGA birth rates, and SGA birth rates tended to reach their lowest values after the onset of the pandemic. The interrupted time series analysis revealed that the pandemic decreased preterm birth, TLBW, and SGA birth rates. In addition, the regression analysis revealed that the pandemic decreased the TLBW, SGA, and artificial fetal mortality rates.

**Conclusions:**

Analyses performed using national data suggested that the pandemic decreased the TLBW and SGA rates in Japan.

**Supplementary Information:**

The online version contains supplementary material available at 10.1186/s12889-024-18905-z.

## Background

Preterm birth, small-for-gestational-age (SGA) birth, and low birth weight (LBW) are major adverse birth outcomes that are risk factors for neonatal and infant mortality [1–3], and these birth outcomes can also affect children’s neurodevelopmental outcomes and body mass index (BMI) [4, 5]. It is known that the LBW rate among singleton births has been decreasing in recent years in Japan (from 8.39% in 2010 to 8.14% in 2019) [6], while the rate of preterm birth has remained relatively constant (approximately 5.6%) [7]. In addition, the fetal mortality rate is another representative outcome of maternal and child health in Japan. Spontaneous and artificial fetal mortality rates have continued to drop over the decades in Japan, while there are more than 10,000 cases of fetal mortality every year even nowadays [8].

The coronavirus disease 2019 (COVID-19) pandemic caused worldwide turmoil in 2020, and it affected trends of multiple health outcomes in Japan, such as the suicide rate and the rate of hospitalization due to cardiovascular diseases [9–11]. In addition, it is known that the pandemic affected the trend in birth outcomes in other countries. Although the results varied from one country to the next, it was suggested that trends in birth outcomes, such as the rates of preterm birth, LBW, and stillbirth changed during the pandemic in certain countries [12–15]. In Japan, there are some studies that investigated trends in birth outcomes, and one study showed that the number of total births significantly decreased in Japan after the pandemic [16]. In addition, there are some studies that investigated trends in adverse birth outcomes and fetal mortality over the decades in Japan using national data [5, 6, 17], while such studies did not evaluate the effect of the pandemic. Moreover, there are some studies that investigated an effect of the pandemic on adverse birth outcomes or threatened preterm labor in Japan [18–23]. In contrast, the sample size used in each study was relatively small, and it is meaningful to evaluate an effect of the pandemic on the adverse outcomes using nationwide national data in Japan.

In this study, we investigated the effect of the onset of the pandemic on trends in adverse birth outcomes and fetal mortality using national data in Japan.

## Methods

### Data and data processing

We used birth data and fetal mortality data from the Vital Statistics in Japan from 2010 to 2022. The individual-level data from the Vital Statistics were provided by Japan’s Ministry of Health, Labour and Welfare on the basis of Article 33 of the Statistics Act in Japan. It is required by law for parents to register their children’s births and fetal mortalities with the local government (municipality) in Japan. Then, each local government sends the data electronically to the Ministry of Health, Labour and Welfare though healthcare center and prefecture, and the data are aggregated by the Ministry. Therefore, data regarding almost all births and fetal mortalities in Japan are included in the Vital Statistics, except for those that were not reported to the local government. Quality check was conducted by the Ministry. Specifically, data on household occupation, sex, birth year, birth month, number of fetuses (multiplicity of births), birth weight, gestational age (weeks and days), wedlock status, parity (maternal past experience of live births and maternal past experience of stillbirths), maternal nationality, and maternal age were used for birth data. In addition, data on the year of death, month of death, number of fetuses, type of fetal mortality, maternal nationality, maternal wedlock status, maternal age, parity (maternal past experience of live births and maternal past experience of stillbirths), gestational age, and household occupation were used for fetal mortality data. The calculation method of gestational age (ultrasound or the last menstrual period) is not stipulated in the survey. The Vital Statistics collects fetal mortality data after the completion of 12 gestational weeks. Therefore, not all miscarriages and induced abortions are included in the fetal mortality statistics. Artificial fetal mortality is defined as the death of fetuses caused by artificial procedures (procedures against fetuses or their belongings as well as the use of labor-inducing drugs), while all other fetal mortality is classified as spontaneous. The sex of fetus was not used for fetal mortality data because it was unknown in many cases.

Preterm birth rate, term low-birth-weight rate (TLBW rate), SGA rate, large-for-gestational age (LGA) rate, spontaneous fetal mortality rate, and artificial fetal mortality rate were used as the outcomes. Only live birth data were used for the analysis of preterm birth, TLBW birth, SGA birth, and LGA birth. In contrast, both live birth and fetal mortality data were used for the analysis of spontaneous and artificial fetal mortality by combining the two types of data. Preterm births are live births that occur at a gestational age of less than 37 weeks. The TLBW rate indicated the rate of births whose birth weight is less than 2,500 g among live births that occur at a gestational age of at least 37 weeks [24]. SGA birth was defined as live births whose birth weight was less than the 10th percentile for each combination of sex, parity, and gestational age (weeks and days), and LGA birth was defined as live births whose birth weight was more than the 90th percentile. These were calculated on the basis of the neonatal anthropometric chart by gestational age in Japan, which was published by the Japan Pediatric Society [25]. Because the anthropometric chart covers only 22–41 completed weeks of gestation, we used only live birth data of those gestational weeks for the analysis of SGA and LGA birth. The spontaneous fetal mortality rate is defined as the number of cases of spontaneous fetal mortality per sum of all the live births and fetal mortality, and the same applies to the artificial fetal mortality rate. Maternal age was classified into 5-year age groups, and maternal nationality was grouped into Japanese and non-Japanese. In addition, a continuous time point variable, which takes 1 in January 2010 and 156 in December 2022, was also created.

To evaluate the effect of the pandemic on birth outcomes, a time point of the onset of the effects of the pandemic should be defined. Similar previous studies in Japan define the starting time of the pandemic as March 2020 or April 2020 in many cases [26–30]. We defined the starting time point as April 2020 because a state of emergency was declared for the first time at this point [31], and self-restraint of social activities spread all over Japan.

### Statistical analysis

We used only singleton births or fetuses for the analysis. We tallied the number of live births and fetal mortality by birth characteristics. In addition, we calculated the preterm birth rate, TLBW rate, SGA rate, LGA rate, spontaneous fetal mortality rate, and artificial fetal mortality rate for each month in the periods, and showed the trends in these outcomes over the years. Then, an interrupted time series analysis was conducted using the monthly time series data. An interrupted time series is often used for evaluating the effect of a public health intervention on health outcomes, and time series data are used in the analysis [32]. Specifically, the autoregressive integrated moving average model with exogenous variables (ARIMAX) was used to evaluate the effect of the pandemic on the outcomes [33, 34]. The rates of birth outcomes were used as outcome variables, and a dummy variable of the effects of the pandemic was included in the model. We changed the orders of autoregressive terms, lags, and moving average terms in the model, and a model whose Akaike information criterion was the lowest was employed. In addition, the seasonality of the data was taken into account by considering 12 months as one season. The coefficient of the effects of the pandemic, its 95% confidence interval (CI), and its p-value were calculated. In addition, ARIMA was used to forecast the values of the outcomes in pandemic periods based on the values obtained during pre-pandemic periods. By comparing the forecasted and actual values, we could infer the pandemic’s effect on the outcomes.

Moreover, the modified Poisson regression model was used to evaluate the effect of the pandemic on these outcomes using individual-level data [35]. In addition to the crude risk ratio, the adjusted risk ratio for the effect on each outcome was calculated. In the analysis, the time point variable, birth month (January, …, December), household occupation, wedlock status, past experience of live births, past experience of stillbirths, maternal nationality, and maternal age group were adjusted in the analysis of the two fetal mortality outcomes, and sex was also adjusted in the analysis of the three live birth outcomes. These explanatory variables (sex, household occupation, wedlock status, past experience of live births, past experience of stillbirths, maternal nationality, and maternal age group) were chosen because they have been shown to be linked to adverse birth outcomes in Japan [36, 37]. Furthermore, birth month was included because adverse birth outcomes exhibit seasonality. The time point variable was also used because most outcomes showed a decreasing or increasing trend over time, and the effect of time trend should be take into account when evaluating the effect of the pandemic. The risk ratio of the effects of the pandemic, robust variance, 95% CI, and p-value were calculated.

The complete-case analysis was conducted for the live birth outcomes and fetal mortality outcomes, respectively. Multiple imputation was also used as a sensitivity analysis of missing data for the regression analysis. Two-sided tests were performed for all analyses, and p-values less than 0.05 were considered statistically significant. All analyses were conducted using R4.1.3 [38], and lmtest [39], sandwich [40], forecast [41], and mice [42] were used as packages. The statistics shown in this study were produced by the authors using the data which were provided by the Ministry of Health, Labour and Welfare in Japan, and they are different from the statistics that were publicized by the Ministry.

## Results

**Fig. 1:**
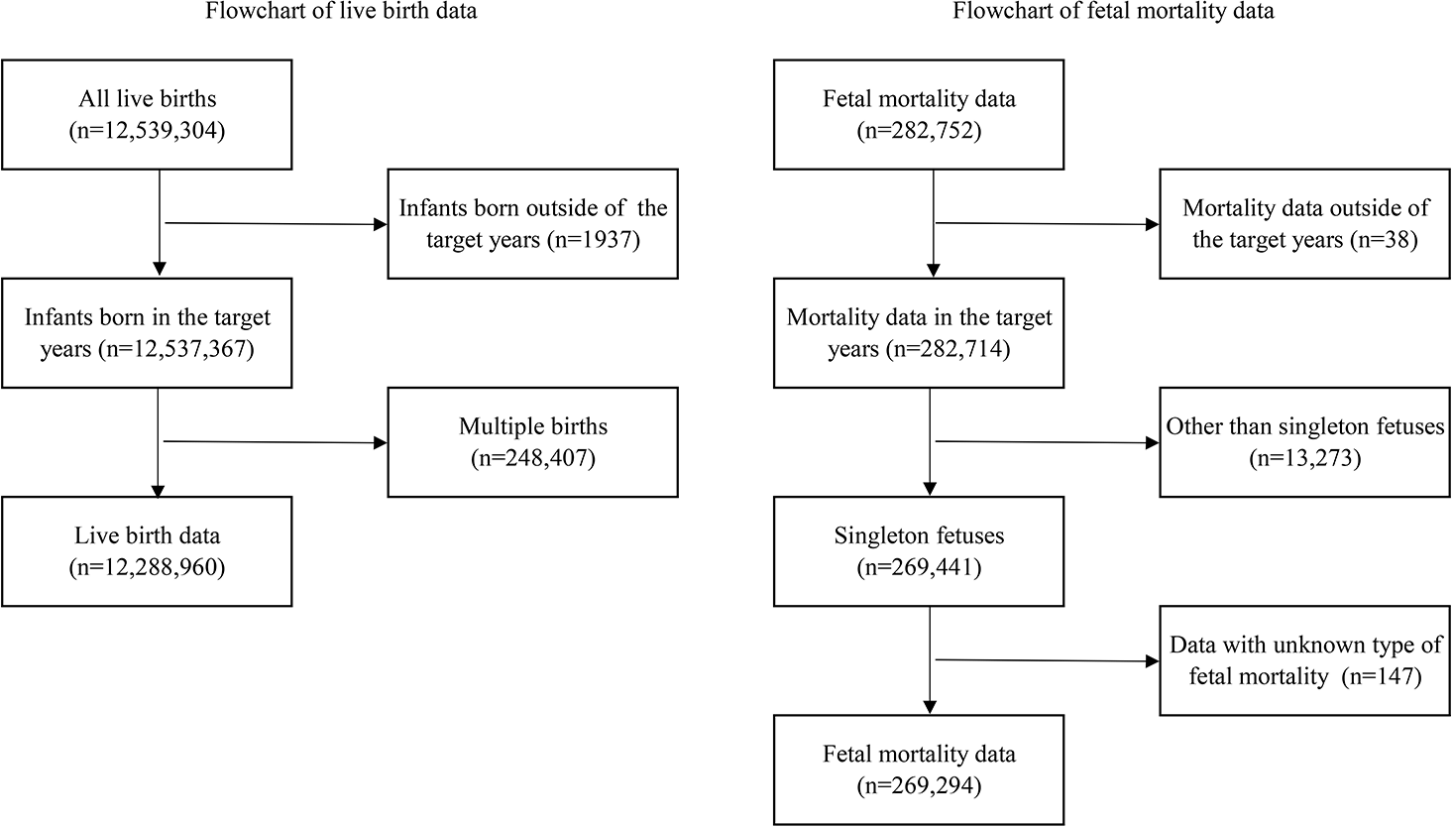
The flowchart of selection of the study population

Figure 1 shows the flowchart of the data selection process. After the selection, the data of 12,288,960 births and 269,294 fetal mortalities were used for the analysis.

Table 1 shows the number (%) of live births and fetal mortality according to birth characteristics. In the analyzed data, the numbers of births and fetal mortality in the post-pandemic period were approximately one-fifth of those in the pre-pandemic period. As shown in Table 1, the data had a low rate of missing values.

**Table 1:** The number (%) of live births and fetal mortality by the birth’s characteristics

Characteristics	Live birth data		Fetal mortality data	
	Before April 2020	April 2020 or later	Before April 2020	April 2020 or later
Total	10,066,937 (100.0)	2,222,023 (100.0)	226,113 (100.0)	43,181 (100.0)
Maternal age group				
Under 20 years	118,544 (1.2)	15,519 (0.7)	22,086 (9.8)	2,485 (5.8)
20–24 years	907,374 (9.0)	165,945 (7.5)	36,109 (16.0)	6,494 (15.0)
25–29 years	2,715,440 (27.0)	583,336 (26.3)	44,040 (19.5)	8,379 (19.4)
30–34 years	3,615,404 (35.9)	802,606 (36.1)	55,393 (24.5)	10,967 (25.4)
35–39 years	2,223,155 (22.1)	520,585 (23.4)	47,937 (21.2)	10,045 (23.3)
40 years or more	486,993 (4.8)	134,024 (6.0)	20,537 (9.1)	4,810 (11.1)
Missing	27 (0.0)	8 (0.0)	11 (0.0)	1 (0.0)
Maternal nationality				
Japanese	9,818,094 (97.5)	2,153,549 (96.9)	219,771 (97.2)	41,317 (95.7)
Non-Japanese	248,843 (2.5)	68,474 (3.1)	6,342 (2.8)	1,864 (4.3)
Past experience of live births				
No	4,766,494 (47.3)	1,035,494 (46.6)	116,635 (51.6)	20,974 (48.6)
Yes	5,300,443 (52.7)	1,186,529 (53.4)	109,478 (48.4)	22,207 (51.4)
Past experience of stillbirths				
No	10,020,203 (99.5)	2,212,708 (99.6)	221,402 (97.9)	42,367 (98.1)
Yes	46,734 (0.5)	9,315 (0.4)	4,706 (2.1)	810 (1.9)
Uncertain	–	–	5 (0.0)	4 (0.0)
Wedlock status				
In wedlock	9,817,729 (97.5)	2,166,031 (97.5)	143,318 (63.4)	30,225 (70.0)
Out of wedlock	249,208 (2.5)	55,992 (2.5)	82,795 (36.6)	12,956 (30.0)
Household occupation				
Farmer	134,454 (1.3)	21,349 (1.0)	2,977 (1.3)	420 (1.0)
Self-employed worker	717,023 (7.1)	156,301 (7.0)	16,862 (7.5)	3,321 (7.7)
Full-time worker 1 ^a^	3,342,392 (33.2)	669,320 (30.1)	69,965 (30.9)	13,243 (30.7)
Full-time worker 2 ^b^	4,466,124 (44.4)	1,068,247 (48.1)	64,148 (28.4)	14,386 (33.3)
Others	858,274 (8.5)	205,212 (9.2)	35,091 (15.5)	6,460 (15.0)
Unemployed	201,595 (2.0)	33,344 (1.5)	21,291 (9.4)	3,059 (7.1)
Missing	347,075 (3.4)	68,250 (3.1)	15,779 (7.0)	2,292 (5.3)
Type of fetal mortality				
Spontaneous	–	–	102,895 (45.5)	20,543 (47.6)
Artificial	–	–	123,218 (54.5)	22,638 (52.4)
Sex				
Male	5,165,510 (51.3)	1,139,337 (51.3)	–	–
Female	4,901,427 (48.7)	1,082,686 (48.7)	–	–
Gestational age				
< 36 weeks	477,113 (4.7)	103,339 (4.7)	–	–
>= 37 weeks	9,586,817 (95.2)	2,118,195 (95.3)	–	–
Missing	3,007 (0.0)	489 (0.0)	–	–
Birth weight				
1499 g or less	60,640 (0.6)	13,062 (0.6)	–	–
1500–2499 g	766,832 (7.6)	163,706 (7.4)	–	–
>=2500 g	9,237,543 (91.8)	2,044,934 (92.0)	–	–
Missing	1,922 (0.0)	321 (0.0)	–	–
Status of SGA and LGA				
SGA	740,533 (7.4)	147,638 (6.6)	–	–
AGA	8,238,181 (81.8)	1,825,095 (82.1)	–	–
LGA	1,059,835 (10.5)	245,981 (11.1)		
<22 or > 41 weeks of gestation	24,086 (0.2)	2,577 (0.1)	–	–
Uncertain	4,302 (0.0)	732 (0.0)	–	–

SGA, small-for-gestational-age; AGA, appropriate-for-gestational-age; LGA, large-for-gestational-age

a Full-time worker 1 means household with a full-time worker in a company whose number of employees is less than 100

b Full-time worker 2 means household with a board member or a full-time worker who does not correspond with the full-time worker 1

**Fig. 2:**
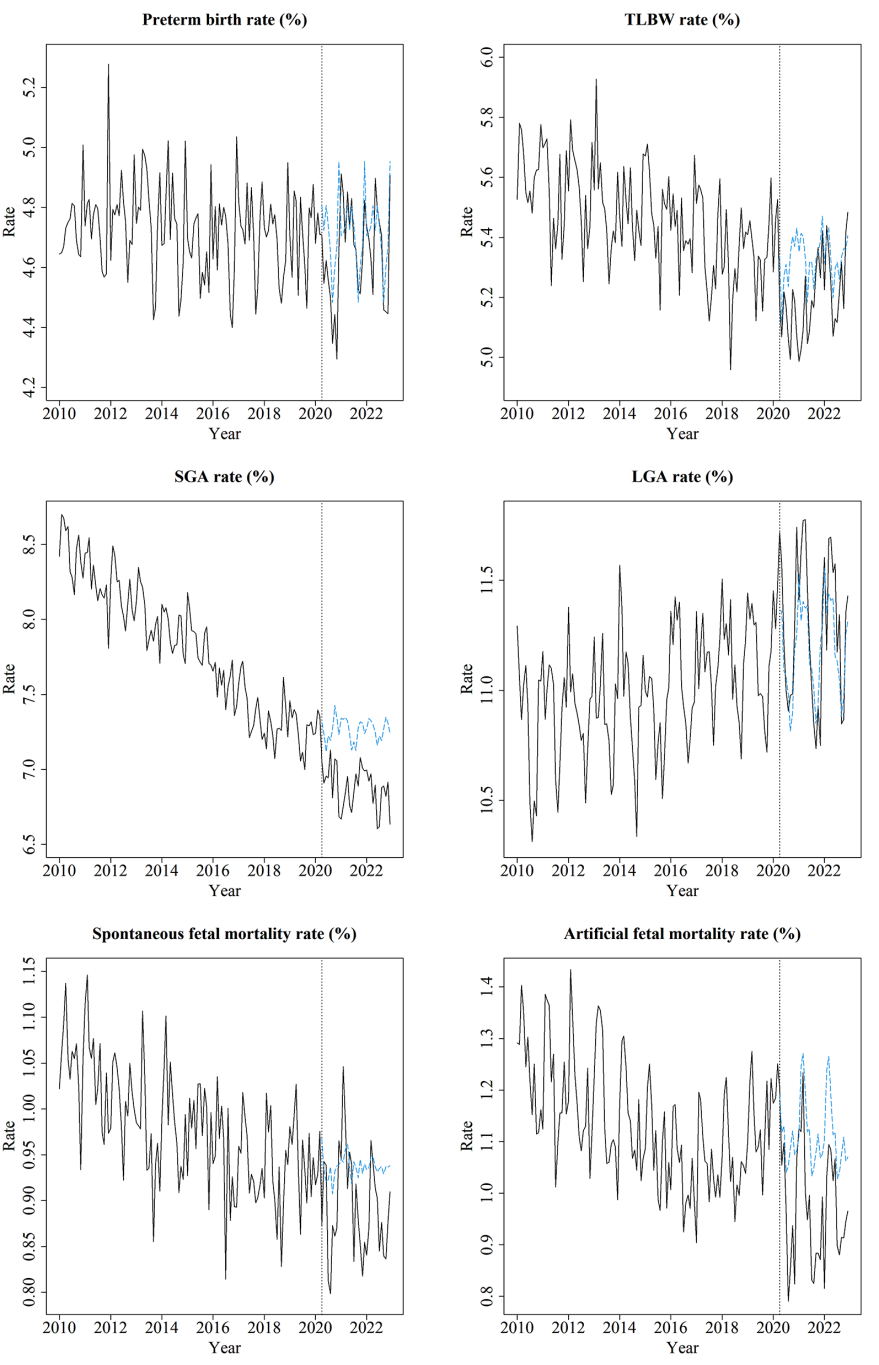
Monthly trend of the adverse outcomes. The vertical dotted line indicates April 2020, when the effects of the pandemic began. The blue dashed line indicates forecasted values, which were calculated from the pre-pandemic periods

Figure 2 shows the monthly trend of adverse outcomes. The preterm birth rate was relatively flat over the years, while it temporarily decreased after the onset of the pandemic. The TLBW rate showed a decreasing trend over the years, whereas it showed an increasing trend from the middle of the pandemic period. The SGA birth rate decreased over the years, reaching its nadir during the pandemic. LGA birth rate showed an increasing trend over the analyzed period. Spontaneous and artificial fetal mortality rates showed a decreasing trend over the years, while an apparent surge in these rates was observed in the middle of the pandemic period. Furthermore, forecasted values in the post-pandemic periods, which were calculated based on the values in the pre-pandemic periods, tended to be higher than the actual values for TLBW rate, SGA birth rate, and artificial fetal mortality rate.

Table 2 shows the result of the interrupted time series analysis showing the effect of the pandemic on the adverse outcomes. The coefficient was significantly lower than 0 for preterm birth, the TLBW, and SGA birth rates, and it indicated that the pandemic had an effect of decreasing these outcomes.

**Table 2:** The result of the interrupted time series analysis showing an effect of the pandemic on the adverse outcomes

Outcome variable	Coefficient (95%CI)	*p*-value
Preterm birth	−0.074 (− 0.125, − 0.024)	0.004
TLBW	−0.194 (− 0.322, − 0.066)	0.003
SGA	−0.225 (− 0.381, − 0.070)	0.004
LGA	0.102 (− 0.002, 0.207)	0.055
Spontaneous fetal mortality	−0.046 (− 0.101, 0.009)	0.100
Artificial fetal mortality	−0.055 (− 0.141, 0.031)	0.212

TLBW, term low birthweight; SGA, small − for − gestational − age; CI, confidence interval

Table 3 shows the result of the regression analysis showing the risk ratio of the pandemic on the adverse outcomes. The crude risk ratios of all the outcomes were statistically significant. In contrast, the adjusted risk ratio was significantly lower than 1 for the TLBW rate (RR [95% CI]: 0.989 [0.981, 0.998]), SGA birth rate (RR [95% CI]: 0.986 [0.979, 0.994]), and artificial fetal mortality rate (RR [95% CI]: 0.980 [0.962, 0.998]), indicating that the pandemic had an effect of decreasing these outcomes.

**Table 3:** The result of the regression analysis showing risk ratio of the pandemic on the adverse outcomes

Outcome variable	Crude analysis		Adjusted analysis	
	RR (95%CI)	*p* − value	RR (95%CI)^a^	*p* − value
Preterm birth	0.982 (0.976, 0.989)	< 0.001	0.997 (0.987, 1.006)	0.471
TLBW	0.950 (0.944, 0.956)	< 0.001	0.989 (0.981, 0.998)	0.019
SGA	0.903 (0.898, 0.908)	< 0.001	0.986 (0.979, 0.994)	< 0.001
LGA	1.050 (1.046, 1.055)	< 0.001	1.006 (1.000, 1.012)	0.054
Spontaneous fetal mortality	0.910 (0.896, 0.924)	< 0.001	1.000 (0.979, 1.021)	0.97
Artificial fetal mortality	0.854 (0.841, 0.866)	< 0.001	0.980 (0.962, 0.998)	0.033

TLBW, term low birthweight; SGA, small − for − gestational − age; LGA, large − for − gestational − age; RR, risk ratio; CI, confidence interval

a Time point, birth month, household occupation, wedlock status, past experience of live births, past experience of stillbirths, maternal nationality, and maternal age group were adjusted for each outcome, and infant’s sex was also adjusted in the analysis of the four live birth outcomes

The Supplementary Table 1 shows the result of the regression analysis showing the risk ratio of the pandemic on the adverse outcomes using multiple imputation. The results were relatively similar to the findings of the complete-case analysis, while a statistically significant effect of the pandemic was not observed for artificial fetal mortality.

## Discussion

This study investigated the effect of the COVID-19 pandemic on adverse birth outcomes and fetal mortality using national data in Japan. As a result, it was suggested that the pandemic decreased the TLBW and SGA birth rates in Japan, regardless of the models that were employed. We discuss possible reasons for the association, implications, and limitations of the findings.

There was a negative association between the COVID-19 pandemic and TLBW and SGA rates. The prevalence of obesity increased in Japan during the pandemic [43], and a similar increase in the prevalence of obesity or weight during the pandemic was also observed in other countries [44, 45]. It has also been reported that staying indoors for a long time increases nutrition intakes for people in another country, and prolonged sedentary behavior might be a cause for BMI changes also among people in Japan [46]. Low BMI is known to be a major risk factor for SGA and LBW among Japanese women [47], and it is possible that an increase in pregnant women’s BMI had a favorable effect on SGA and TLBW rates in Japan. In contrast, the reason why the TLBW rate showed an increasing trend in the middle of pandemic period is uncertain. Although it might be just a natural fluctuation, it might reflect a gradual lifestyle change by ease of restrictions from the pandemic. Actually, ease of restrictions from the pandemic is shown to be associated with increased physical activity among Japanese adolescents [48].

Regarding artificial fetal mortality, the results differed depending on the models. Specifically, the results of analyses performed using individual-level data with multiple imputation and the time series analysis did not show a significant effect of the pandemic. In contrast, the coefficients of the interrupted time series analysis indicated a decreasing effect, and the rate tended to take the lowest levels in the decade after the pandemic’s onset. Therefore, it is possible that there were some negative effects of the pandemic on the artificial abortion rate. A pandemic effect of decreasing the number of abortions and the fertility rate was observed in Mexico [49], and the fertility rate also decreased during the stay-at-home period [50]. A decrease in the rates of unwanted pregnancy among single and adolescent women was pointed out as a possible factor for a decrease in the number of abortions [49]. Factors such as reductions in the amount of face-to-face social interactions because of the quarantine due to the pandemic may have affected the trend in the rate of unplanned pregnancies in Japan.

Regarding the rate of preterm birth, no significant effect of the pandemic was shown in the regression analysis, while a significant effect was observed in the interrupted time series analysis. The results of the effect of the COVID-19 pandemic on preterm birth varied from one country to the next [51–54], and many studies have reported that there was no association between the pandemic and preterm birth.

The results of this study suggested that some of the adverse birth outcomes may have been affected by the COVID-19 pandemic. However, there is a paucity of currently available post-pandemic data and further analyses using data from later years needs to be conducted to verify whether the pandemic had affected these birth outcomes. In addition, mechanisms for the associations between the pandemic and adverse birth outcomes are uncertain, and a study investigating an association of the onset of COVID-19 with other outcomes, such as pregnancy complications and BMI of pregnant women needs to be conducted in Japan. Utilization of antenatal care is another factor that can influence adverse birth outcomes. In other countries, the use of antenatal care was affected by the pandemic [55, 56]. It will be meaningful to investigate the trends in antenatal care utilization before and after the pandemic in Japan.

This study had some limitations. First, the rate of adverse outcomes can be influenced by multiple factors, such as changes in physical characteristics and medical practices, and it is possible that factors other than the pandemic affected the trends. In addition, data on lifestyles and physical characteristics of parents, such as smoking status, utilization of antenatal care, BMI, and physical activity were not available in the Vital Statistics. In contrast, the main strength of this study is that we used national data in Japan, and the study population covers all of the birth and fetal mortality data in that nation.

## Conclusions

This study investigated the effect of the onset of the pandemic on the trend of adverse birth outcomes and fetal mortality using national data in Japan. The result of the interrupted time series analysis showed that the pandemic had a decreasing effect on preterm birth, the TLBW, and SGA rates. In addition, the result of the regression analysis showed that the pandemic had a decreasing effect on the TLBW, SGA, and artificial fetal mortality rates. It was suggested that the pandemic affected the rates of some birth outcomes.

## Electronic supplementary material

Below is the link to the electronic supplementary material.

Supplementary Material 1

## Data Availability

The data that support the findings of this study are available from the Ministry of Health, Labour, and Welfare in Japan but restrictions apply to the availability of these data, which were used under license for the current study, and so are not publicly available. Data are however available from the authors upon reasonable request and with permission of the Ministry of Health, Labour, and Welfare in Japan.

## Abbreviations

ARIMAX: autoregressive integrated moving average model with exogenous variables
BMI: body mass index
CI: confidence interval
COVID-19: coronavirus disease of 2019
LGA: large-for-gestational-age
RR: risk ratio
SGA: small-for-gestational-age
TLBW: term low birthweight
