# Publisher Correction: Effects of the COVID-19 pandemic on the rates of adverse birth outcomes and fetal mortality in Japan: an analysis of national data from 2010 to 2022

**DOI:** 10.1186/s12889-024-19870-3

**Published:** 2024-09-18

**Authors:** Tasuku Okui, Naoki Nakashima

**Affiliations:** Medical Information Center, Kyushu University Hospital, Maidashi3-1-1 Higashi-Ku Fukuoka City Fukuoka Prefecture, Fukuoka City, 812-8582 Japan

**Publisher Correction: BMC Public Health 24, 1430 (2024)**

**https://doi.org/10.1186/s12889-024-18905-z**

In the original publication of this article the author names were in the incorrect order. In addition: an incorrect version of figure 2 was published. The incorrect and correct information is shown in this correction article, the original has been updated.

**Incorrect**

Okui Tasuku & Nakashima Naoki

**Correct**

Tasuku Okui & Naoki Nakashima

**Incorrect figure 2**



**Correct figure 2**


