# Supplementary material for: Effects of the COVID-19 pandemic on the rates of adverse birth outcomes and fetal mortality in Japan: an analysis of national data from 2010 to 2022
Source: BMC Public Health. 2024 May 28;24:1430. doi: 10.1186/s12889-024-18905-z (PMC11134758; doi:10.1186/s12889-024-18905-z)
Supplement: Supplementary file 1 — Supplementary Material 1 [file 12889_2024_18905_MOESM1_ESM.pdf]

Supplementary table 1. The result of the regression analysis showing risk ratio of the pandemic on the adverse outcomes using multiple imputation.

	Crude analysis		Adjusted analysis	
Outcome variable	RR (95%CI)	p-value	RR (95%CI)[a]	p-value
Preterm birth	0.981 (0.975, 0.988)	<0.001	0.997 (0.988, 1.006)	0.527
TLBW	0.949 (0.943, 0.955)	<0.001	0.988 (0.980, 0.997)	0.007
SGA	0.902 (0.897, 0.907)	<0.001	0.986 (0.979, 0.994)	<0.001
LGA	1.050 (1.046, 1.054)	<0.001	1.005 (0.999, 1.011)	0.077
Spontaneous fetal mortality	0.907 (0.894, 0.921)	<0.001	1.000 (0.979, 1.020)	0.983
Artificial fetal mortality	0.835 (0.823, 0.847)	<0.001	0.987 (0.969, 1.005)	0.153

TLBW, term low birthweight; SGA, small-for-gestational-age; LGA, large-for-gestational-age; RR, risk ratio; CI, confidence interval

a Time point, birth month, household occupation, wedlock status, past experience of live births, past experience of stillbirths, maternal nationality, and maternal age group were adjusted for each outcome, and infant's sex was also adjusted in the analysis of the four live birth outcomes
